# Editorial: Music and AI

**DOI:** 10.3389/frai.2021.651446

**Published:** 2021-02-11

**Authors:** Alexandra Bonnici, Roger B. Dannenberg, Steven Kemper, Kenneth P. Camilleri

**Affiliations:** ^1^ Department of Systems and Control Engineering, University of Malta, Msida, Malta; ^2^ School of Computer Science, Carnegie Mellon University, Pittsburgh, PA, United States; ^3^ Mason Gross School of the Arts, Rutgers, The State University of New Jersey, New Brunswick, NJ, United States

**Keywords:** music composition, expressive playing, music recommendation, sight-reading, music interfaces, music and mental health, artificial inteligence, machine learning

Computer algorithms have been shaping the music scene since the 1950s. Artificial intelligence, machine learning and computational methods have left their mark not only on the way that music is composed and performed but also on the adoption of new musical notations; different music learning approaches as well as in different marketing strategies which change the way music is consumed.

## AI in the Production of New Music

Computational techniques have been used in a variety of ways for the creation and production of musical compositions with this field predating even digital music synthesis. Algorithmic music composition techniques range from the use of stochastic processes to create music based on random events to learning-based approaches. The paper “**Computational Creativity and Music Generation Systems: an Introduction to the State of the Art**” (Carnovalini and Roda) reviews work extending over six decades, organizing their presentation by methods, ranging from Markov chains to deep networks, and offering a set of open challenges. An extensive bibliography is included. Just as the techniques used to generate the music are varied, so too is the style of music generated, from the creation of written scores to musical accompaniment. “**Evolving Musical Sight Reading Exercises Using Expert Models**” (Pierce et al.) presents a novel evolutionary algorithm for generating monophonic sight-reading exercises in the Western art music tradition. Drawing on expert models of published sight-reading exercises, the evolutionary process draws on six fitness measures to create new exercises designed for specific grade levels of musical instruction. These include target note lengths, target rest lengths, allowable lengths, target intervals, allowable intervals, and melody shape. “**On the Adaptability of Recurrent Neural Networks for Real-Time Jazz Improvisation Accompaniment**” (Kritsis et al.) describes the basic implementation of an artificial jazz accompanist system that provides real-time accompaniment to a human musician soloist, based on a given harmonic description of lead sheet chord symbols. Recurrent Neural Networks are employed both for modeling the predictions of the artificial agent and for modeling the expectations of human intention. Fuzzy logic is a branch of AI that is often overlooked in this age of big data and neural networks. Nevertheless, fuzzy logic can be a powerful tool for modeling and learning music information expressed as signals, parameters or symbols. Fuzzy logic offers a useful framework for expressing models that can assist learning from less data. “**Creating Music with Fuzzy logic**” (Cadiz) offers an introduction to this field and describes a software toolkit created for composition and real-time music applications.

## AI to Create Expressive Music

Music must be performed expressively to be engaging. Computational musical expression systems learn expressive performance models from examples of human performances to adapt these to the music at hand. Expressive music performance thus alters the "mechanical" or literal performances implied by discrete music notation symbols into more nuanced realizations with alterations in timing, dynamics, timbre, vibrato and other details. “**A Dynamic Representation Solution for Machine Learning-Aided Performance Technology**” (Palamara and Deal) considers the use of AI techniques to interpret discrete dynamic values, such as *p*, *mp*, *f* and *ff* to control parameters that can adapt to performance context.

## Easy-To-Use Music Interfaces

As machine learning makes its way from the worlds of science, technology and commerce to the arts, there is a need for easy-to-use systems and interfaces that can be applied directly by artists, composers and performers. “**Evaluating the Usability of an API for Rapid Prototyping Music Technology with Interactive Machine Learning**” (Bernardo et al.) considers the problem of supporting designers of creative software projects with tools for machine learning. The study offers insights into both the design of machine learning frameworks and evaluation strategies. Another application of AI is toward intelligent instruments that adapt to or enrich human performance gestures. **Understanding Musical Predictions with an Embodied Interface for Musical Machine Learning**” (Martin et al.) implements a purposefully simple musical instrument with just one input, a lever controlling pitch, and an internal sequence-prediction algorithm based on a recurrent neural network and trained on human performances. The study sheds light on how humans interact with predictive gestural interfaces. In “**Automated Page Turner for Musicians**” (Tabone et al.), the authors describe using eye-gaze tracking to enable hands-free page turning, employing Kalman filtering to balance the music-reading model and the noisy eye-gaze data, thus obtaining stable and reliable page-turning.

## AI in Marketing of Music

Music providers are also using AI to learn the musical preferences of consumers to provide customised playlists based on listening patterns. “**Listener Modeling and Context-aware Music Recommendation Based on Country Archetypes**” (Schedl et al.) considers how music preferences are shaped by the country of the listener. The study uses unsupervised learning to suggest nine archetypes or clusters of listening preferences and shows that recommendation systems can be enhanced by using country information.

## Music and Healthcare

Music has many implications for health care, and computer-generated music is of special interest due to the possibility of making music for specific functions or according to particular therapeutic constraints. “**On the use of AI for Generation of Functional Music to Improve Mental Health**” (Williams et al.) uses machine learning to create music targeting a specific physiological response. This work suggests a new direction for the evaluation of music generation systems as well as future applications such as games and health care.

